# Cave reservoir characterization method driven by GA-KPCA and geological knowledge

**DOI:** 10.1371/journal.pone.0344440

**Published:** 2026-03-19

**Authors:** Wenbo Ren, Huaxin Chen, Ruiqi Wang, Tao Zhang, Linjun Li

**Affiliations:** 1 Yangtze University, Wuhan, China; 2 Sinopec Northwest Oilfield Branch, Urumqi, Xinjiang, China; 3 College of Geophysics, Chengdu University of Technology, Chengdu, China; Ministry of Education, MOROCCO

## Abstract

This paper presents a novel method for cave reservoir characterization based on the Genetic Algorithm (GA) and Kernel Principal Component Analysis (KPCA), aimed at improving the precision of reservoir characterization through adaptive multi-attribute fusion. Sensitive seismic attributes are first extracted using geophysical algorithms and their correlations are analyzed based on geological interpretation. Initial attribute weights are then determined scientifically, ensuring reliable geological input for the fusion process. KPCA, with its strong nonlinear analysis capabilities, is used for efficient clustering and feature extraction of complex cave data, while GA optimizes KPCA’s key bandwidth parameter to enhance search efficiency. The GA-KPCA method was validated using both synthetic cave model data and real carbonate rock field data in Tarim Basin, demonstrating significant advantages over traditional methods. The results indicate that the proposed approach effectively addresses the limitations of existing techniques, improving the reservoir identification success rate by approximately 33%, and offering an innovative and efficient solution for cave reservoir exploration and development. This method not only contributes to the advancement of cave reservoir characterization but also provides valuable theoretical and practical insights for future research in the field.

## 1. Introduction

Cave reservoirs, as a unique and significant type of reservoir architecture, hold a pivotal position in global oil and gas exploration and development [1]. Their complex cavern structures provide distinctive spaces for hydrocarbon storage and migration. This significant exploration potential has attracted widespread industry attention. Accurate and detailed characterization of cave reservoir features is essential for understanding formation mechanisms and distribution patterns, as well as for the subsequent formulation of efficient development strategies [2].

For a long time, researchers have been committed to exploring effective methods for characterizing cave reservoirs [3]. Among numerous attempts, methods based on seismic attribute analysis and various inversion techniques have found extensive application.

Using seismic attributes for cave reservoir characterization mainly involves analyzing attributes such as amplitude, frequency, and phase carried by seismic waves propagating underground to infer the location, scale, shape, and internal fillings of caverns [4]. For example, strong amplitude anomalies may imply the existence of caverns, while changes in frequency can help determine the nature of fluids within the caverns [5]. However, single-attribute methods have significant limitations. Subsurface geological complexity limits the information provided by any single attribute, preventing a comprehensive reflection of reservoir characteristics. This leads to uncertain characterization outcomes that fail to satisfy stringent industry accuracy requirements.

Inversion methods, on the other hand, infer the physical parameters of underground geological bodies (such as impedance) from received seismic signals to depict cave reservoirs. Although inversion techniques can provide richer underground information to some extent, they also encounter numerous implementation challenges [6]. First, inversion processes often rely on simplified geological assumptions. Actual cave conditions are complex and variable, meaning these assumptions frequently deviate from reality and affect accuracy [7]. Second, inversion problems are inherently multi-solution. Interference from data noise and observation systems makes obtaining a unique, accurate solution difficult, diminishing reliability [8].

It is worth noting that although different seismic attributes have their own limitations, each has unique advantages in characterizing certain specific aspects of cave reservoirs [9]. For example, amplitude attributes are sensitive to the scale and boundaries of caverns, allowing for a rough outline of the cavern shapes [10]. Frequency attributes have certain advantages in identifying the nature of fillings within caverns, which can help distinguish whether the caverns contain hydrocarbons, water, or other substances [11]. Phase attributes play an important role in identifying discontinuities in strata, thereby revealing the contact relationships between caverns and surrounding formations [12].

Multi-attribute fusion integrates information from various attributes to provide a comprehensive and accurate characterization [13]. Compared with single-attribute methods, it significantly improves the precision and reliability of reservoir characterization, offering more robust data support for subsequent exploration and development. For instance, by fusing amplitude, frequency, and phase attributes, it is possible to more accurately determine key information about cavern locations, scales, shapes, and fluid properties within the caverns. However, practical applications face challenges. Attribute weight determination often lacks a scientific basis, relying instead on subjective settings or simple statistical approaches. This prevents weight allocation from reflecting the actual contribution of each attribute to the characterization [14]. In addition, some multi-attribute fusion algorithms have low computational efficiency when dealing with large-scale, high-dimensional data, and are prone to getting trapped in local optima rather than achieving a globally optimal fusion result. This, to some extent, limits the application scope and effectiveness of the method [15].

Among various multi-attribute fusion methods, the clustering method based on Kernel Principal Component Analysis (KPCA) has demonstrated significant potential in the characterization of cave reservoirs due to its excellent nonlinear feature extraction and data dimensionality reduction capabilities [16]. KPCA maps the original data from a low-dimensional space to a high-dimensional feature space through a nonlinear mapping, allowing for more effective extraction of principal component information in the high-dimensional space. This enables efficient clustering and feature extraction of complex data [17].

In the context of cave reservoir characterization, KPCA can integrate multiple seismic attributes to extract principal components that reflect the essential characteristics of cave reservoirs, thereby achieving precise classification and characterization. However, the effectiveness of the KPCA method largely depends on the selection of its parameters [18]. Improper parameter choices can lead to ineffective feature extraction and consequently affect the accuracy of reservoir characterization.

Moreover, during the multi-attribute fusion process, different attributes exhibit varying sensitivities to cave reservoirs. Therefore, their initial weights in the fusion should not be identical [19]. For example, in some applications, weighted KPCA methods have been proposed to address the issue of differing attribute sensitivities by assigning different weights to attributes based on their importance. This approach can potentially enhance the performance of KPCA in characterizing complex geological structures such as cave reservoirs.

To address the challenges of parameter selection in KPCA and the scientific determination of initial weights for attributes, this paper introduces a new method for characterizing cave reservoirs, integrating Genetic Algorithm (GA) with Kernel Principal Component Analysis (KPCA) and geological knowledge. This method leverages the adaptive optimization capabilities of GA, the nonlinear feature extraction of KPCA, and the guiding role of geological knowledge, thereby enhancing the effectiveness of multi-attribute fusion.

The GA-KPCA method optimizes the parameters of KPCA through genetic operations such as selection, crossover, and mutation, which helps in finding the optimal parameters for KPCA. Meanwhile, the initial weights of different attributes are determined based on their sensitivities to cave reservoirs, which are inferred from logging interpretation conclusions. This approach ensures that attributes with higher sensitivity to reservoir characteristics are given more weight in the fusion process, improving the accuracy of reservoir characterization.

To validate the effectiveness and feasibility of the proposed method, both synthetic model data and real field data were used for algorithm testing. The results indicate that compared with traditional methods, the GA-KPCA method provides a more accurate and comprehensive characterization of cave reservoirs. It effectively overcomes the limitations of existing methods, offering a novel and efficient technical means for the exploration and development of cave reservoirs.

## 2. Theory

This section introduces the basic principles of the commonly used seismic attributes, Kernel Principal Component Analysis (KPCA), and Genetic Algorithm (GA) for cave reservoir characterization. It concludes by presenting the implementation process of the proposed method in this paper and its specific contributions.

### 2.1. Seismic attribute method

Seismic attributes are parameters extracted from seismic data that can reflect subsurface geological characteristics. They are crucial for characterizing cave reservoirs and can be used to identify the location, scale, shape, and internal fillings of caverns. For cave reservoirs, commonly used sensitive seismic attributes include instantaneous amplitude, sweet spots, and structural tensors.

The calculation of instantaneous amplitude is typically achieved using the Hilbert transform. For a given seismic signal s(t), its analytic signal z(t) is composed of the original signal s(t) and its Hilbert transform $\begin{array}{c} H\left[s\left(t\right)\right] \end{array}$, that is, $\begin{array}{c} z\left(t\right)=s\left(t\right)+iH\left[s\left(t\right)\right]) \end{array}$, the instantaneous amplitude A(t) is the modulus of the analytic signal:


$$A\left(t\right)=\sqrt{s{\left(t\right)}^{\text{2}}+H{\left[s\left(t\right)\right]}^{\text{2}}}$$ (1)

The sweet spot attribute D is used to identify high-potential hydrocarbon areas within reservoirs. Physically, it is a composite indicator derived from the product of the instantaneous amplitude and the reciprocal of the root mean square frequency. This attribute is calculated based on seismic amplitude and frequency information:


D=A(t)×f(t) (2)

Wheref(t) represents the instantaneous frequency of seismic signal s(t). In carbonate reservoir characterization, the sweet spot attribute is particularly effective because it highlights the combined response of strong wave impedance contrasts and frequency attenuation.

Structure tensor attributes are used to describe the local structural characteristics of seismic data and are important for identifying the boundaries and morphology of caves. Let the partial derivatives of the seismic data in the (x) and (y) directions be denoted as $\frac{\partial s}{\partial x}$ and $\frac{\partial s}{\partial y}$, then the structure tensor attribute T can be expressed as:


$$\begin{array}{c} T=\left[\begin{array}{cc} @cc@{\left(\frac{\partial s}{\partial x}\right)}^{\text{2}} & \frac{\partial s}{\partial x}\frac{\partial s}{\partial y}\\ \frac{\partial s}{\partial x}\frac{\partial s}{\partial y} & {\left(\frac{\partial s}{\partial y}\right)}^{\text{2}} \end{array}\right] \end{array}$$ (3)

To enhance the stability and continuity of the tensor, Gaussian-weighted smoothing can be applied, followed by eigenvalue decomposition. This process yields the energy in the principal direction $\lambda_{\text{1}}$ and the energy perpendicular to the principal direction $\lambda_{\text{2}}$. Under normal conditions $\lambda_{\text{1}}\geq \lambda_{\text{2}}$, in this paper, we select $\lambda_{\text{2}}$ to characterize the cave structures.

### 2.2. KPCA

Kernel Principal Component Analysis (KPCA) is a powerful nonlinear principal component analysis method with widespread applications in various fields. The core idea of KPCA is to map the original data from a low-dimensional input space to a high-dimensional feature space F [20] through a nonlinear mapping function. In this high-dimensional feature space, the data may exhibit better linear separability or more clearly reveal the intrinsic structure of the data.

Assuming there is a set of raw data $\text{X = }\;\;\{\;\;\;x_{1},x_{2},\cdots ,x_{n}\}$, which are in a low-dimensional space. Through a nonlinear mapping φ:x→φ(x),they are mapped into a high-dimensional feature space F, and then principal component analysis is carried out in this high-dimensional space, First, calculate the covariance matrix C in the feature space, its expression is:


$$C=\frac{\text{1}}{n}\sum_{i=1}^{n}\varphi (x_{i})\varphi (x_{i}{)}^{T}$$ (4)

The covariance matrix C can reflect the distribution and variation of the data in the high-dimensional space. To solve for the eigenvalues and eigenvectors of the covariance matrix C, we introduce the kernel function $K(x_{i},x_{j})$. The role of the kernel function is to cleverly avoid the complex calculations directly in the high-dimensional space, It satisfies $K(x_{i}\text{,}x_{j})=j(x_{i}{)}^{T}j(x_{j})$. By solving the eigenvalue problem of the n×n matrix K, we can obtain the corresponding eigenvectors α. Based on these results, the principal components z in the high-dimensional feature space can be expressed as:


$$z=\sum_{i=1}^{n}\alpha_{i}K(x,x_{i})$$ (5)

This is the fundamental principle of KPCA. It performs principal component analysis in a high-dimensional space via a kernel function, thereby achieving feature extraction and dimensionality reduction of the original data [21]. In KPCA, the choice of the kernel function is crucial, as it determines the mapping of the data into the high-dimensional space [22]. In this paper, we employ the Gaussian function as the kernel function, also known as the Radial Basis Function (RBF) for three key reasons specific to cave reservoir characterization: First, the RBF kernel’s local sensitivity and nonlinear mapping capability are particularly effective at capturing the complex geometries and heterogeneous features of cave reservoirs. Second, its distance-based formulation naturally highlights the contrast between cave structures and surrounding formations. Third, the single γ parameter provides sufficient flexibility while avoiding overfitting common in more complex kernels. Its expression is:


$$K(x_{i}\text{,}x_{j})=exp(-\frac{{\|x_{i}-x_{j}\|}^{\text{2}}}{2\sigma^{\text{2}}})$$ (6)

Here, $\|x_{i}-x_{j}\|$ represents the Euclidean distance between samples, which measures the spatial proximity between two samples. σ is the bandwidth parameter of the Gaussian kernel function, and this parameter has a significant impact on the performance of the kernel function [23]. A smaller value narrows the scope of the Gaussian kernel function, meaning that only samples that are close in distance will have a significant correlation. In contrast, a larger value widens the scope of the kernel function, resulting in correlations between more samples [24]. In practical applications, the value of σ needs to be adjusted according to the specific characteristics of the data and the requirements of the task. Typically, methods such as cross-validation are used to determine its optimal value to achieve the best KPCA effect. In this paper, we use GA to adaptively simulate this process [25].

### 2.3. GA

GA (Genetic Algorithm) is an optimization algorithm based on the theory of biological evolution. It searches for the optimal solution by simulating the process of natural selection and genetic operations [26]. The search process can be divided into the following steps: encoding, selection, crossover, and mutation.

In the encoding step, the solution to the problem is encoded into a chromosome, with common encoding methods including binary encoding and real-number encoding. For example, for the parameter vector to be optimized $x=(x_{\text{1}},x_{\text{2}},\cdots ,x_{m})$, each parameter x_i_ can be encoded into a binary string or a real number. In this paper, the bandwidth parameter is encoded, with its value range set to [0.1,10]. Subsequently, a fitness function is constructed, which is used to evaluate the quality of each chromosome. In this paper, the fitness function is formulated as a minimization problem with geological significance that integrates the interpretation error:


$$F=\frac{\text{1}}{L+\varepsilon }$$ (7)

Where $L={\left({\text{y}}_{\text{int}}-{\text{y}}_{\text{pred}}\right)}^{\text{2}}$; Here, ${\text{y}}_{\text{int}}$ is the reservoir interpreted by logging, and ${\text{y}}_{\text{pred}}$ represents the reservoir predicted by applying a specific threshold $T_{f}$ to the fused attribute. The selection of $T_{f}$ is critical; it is determined by analyzing the probability density distribution of the fused principal components and aligning it with the interpreted cave scale from pseudo-wells. To ensure the optimization is not overly sensitive to a single value, ϵ is a small regularization constant to avoid division by zero. The above process page reflects the guiding role of geological knowledge throughout the entire optimization process.

Further selection is based on the fitness of the chromosomes, with those having higher fitness having a greater probability of being selected. Taking the roulette wheel selection as an example, the probability $p_{i}$ of each chromosome being selected is:


$$p_{i}=\frac{F(x_{i})}{\sum_{j=1}^{n}F(x_{j})}$$ (8)

Subsequently, crossover operations are performed on the selected chromosomes to generate new chromosomes. For example, in single-point crossover, a crossover point is randomly chosen, and the genetic segments of two parent chromosomes are swapped at this point [27]. Since different bandwidth parameters can affect the extraction and integration of multi-attribute features by KPCA, the new parameter combinations generated through crossover operations may, in subsequent fitness evaluations, identify bandwidth parameters that better align with geological understanding. This, in turn, optimizes the multi-attribute fusion effect [28].

Finally, genetic mutations are introduced to the chromosomes with a certain probability. For instance, this could involve randomly [flipping a bit in a binary string or applying a small perturbation to a real-valued parameter [29]. By introducing mutations, the genetic algorithm can avoid prematurely falling into local optima. This helps in discovering bandwidth parameters that better meet the consistency requirements with geological understanding and enhances the performance of KPCA in multi-attribute fusion for cave reservoirs [30].

### 2.4. The procedure of GA-KPCA method

The proposed GA-KPCA method follows the following technical workflow: First, the seismic data of the cave reservoir to be interpreted is input. The input seismic data is processed to calculate various seismic attributes, which characterize the cave reservoir from different perspectives and contain rich geological information. Then, at least two attributes that are more sensitive and important for subsequent analysis and modeling are selected. These sensitive attributes have a stronger correlation with geological features and can more effectively reflect the information of the cave reservoir. This process is subjective. Further, a correlation analysis is conducted between these sensitive attributes and the reservoir thickness interpreted from well logging. The shared degree of different attributes in characterizing the reservoir is deeply analyzed, and the attributes are weighted and ranked according to their correlation to determine the initial weights of the input attributes. To quantitatively determine the initial weights, the Pearson Correlation Coefficient (r) is calculated to measure the linear relationship between each seismic attribute and the well-log interpreted reservoir parameters. The coefficient is defined as:


$$r=\frac{\sum_{i=1}^{n}(X_{i}-\overset{―}{X})(Y_{i}-\overset{―}{Y})}{\sqrt{\sum_{i=1}^{n}(X_{i}-\overset{―}{X}{)}^{\text{2}}\sum_{i=1}^{n}(Y_{i}-\overset{―}{Y}{)}^{\text{2}}}}$$ (9)

Where $X_{i}$ represents the seismic attribute values, $Y_{i}$ denotes the reservoir thickness from well-log interpretation, $\overset{―}{X}$ and $\overset{―}{Y}$ are their respective mean values, and n is the number of vertical sampling points. The resulting r values are directly utilized as the initial weights, providing a statistically grounded and reproducible initialization for the subsequent optimization. This process is objective. The above processes are constrained by geological prior information, which endows the fusion process with high geological significance. This is referred to as geologically knowledge-driven in this paper.

Subsequently, iterative optimization of the fusion parameters (bandwidth parameters) is carried out. First, the relevant parameters of the genetic algorithm need to be initialized, including population size, crossover probability, mutation probability, etc. These hyperparameters are selected based on empirical testing to effectively balance global search diversity and convergence speed. Simultaneously, the initial weights determined in the knowledge-driven part serve as the initial conditions for the genetic algorithm. Then, the fitness of each individual, representing bandwidth parameter combinations, is evaluated using a function consistent with geological knowledge. The roulette wheel selection strategy is adopted, where individuals with higher fitness have a greater probability of producing the next generation. This ensures that optimal parameter combinations are retained and passed on..

Crossover operations are performed on the selected parent individuals. New offspring individuals are generated by exchanging parts of the genes (i.e., the encoding of bandwidth parameters), providing more possibilities for searching for better solutions. Meanwhile, random mutations are introduced with a specific probability to maintain population diversity and prevent premature convergence to local optima. Finally, whether the termination conditions are met is judged. If the termination conditions are met, the process proceeds to the next step; otherwise, it returns to the fitness calculation step to continue iterative optimization. The termination condition set in this paper can be reaching the preset number of iterations.

When the termination condition is met, the individual with the highest fitness from the final generation is selected. The bandwidth parameters corresponding to this individual are deemed optimal. Subsequently, Kernel Principal Component Analysis (KPCA) is performed using the determined optimal bandwidth parameters to fuse the previously selected sensitive attributes. The final GA-KPCA fusion integrates information from multiple seismic attributes, ensuring the results accurately reflect cave reservoir characteristics. This provides more effective data support for subsequent characterization and analysis of the cave reservoir.

**Fig 1:**
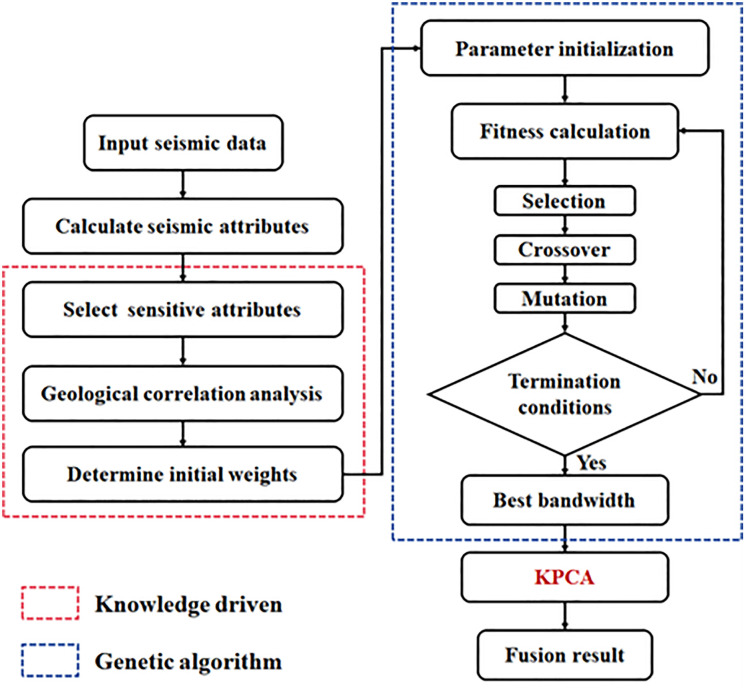
The workflow of the proposed method.

The technical workflow diagram of the proposed method is shown in Fig 1. The contributions of the proposed method are summarized as follows:

1. Determining the initial weights based on geological knowledge has enabled a joint knowledge-data driven approach, endowing the fusion results with high geological significance;
2. Utilizing GA for efficient parameter optimization makes the proposed method more objective and intelligent;
3. Attribute fusion based on KPCA has enhanced the robustness and reliability of the fusion results.

## 3. Examples

This section demonstrates the performance of the proposed knowledge-driven GA-KPCA fusion method. The feasibility and effectiveness of the proposed method are verified using a model dataset and a field carbonate cave reservoir example. In these examples, input attributes follow a standardized normalization workflow to ensure consistency. The optimization is executed based on the fixed parameters and search ranges described in previous sections.

### 3.1. Cave model data

**Fig 2:**
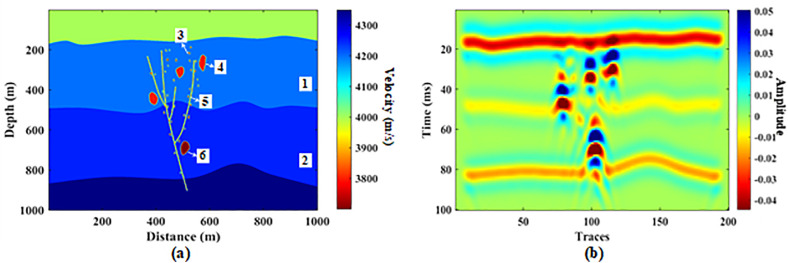
Model data example: (a) geological model and (b) synthetic seismic data.

We first utilize a model dataset to verify the feasibility of the proposed method. As shown in Fig 2a, we designed a geological model with four cave reservoirs, with both the vertical depth and horizontal distance of the model being 1000 m. The velocities of the reservoirs were randomly set between 3700 and 3900 m/s. The background formations have certain gradient variations, and the properties of the formations in different sedimentary periods also differ, with the formation velocities set between 4000 and 4350 m/s (Table 1). To make the model more realistic, we added geological elements such as undulating formations, faults of different scales, and small-scale pore bodies. Additionally, to endow the model with geological significance, we did not use theoretical wavelets for forward modeling but extracted seismic wavelets from real seismic data to conduct this process. After wavefield simulation and time migration, the resulting synthetic seismic data are shown in Fig 2b. It can be seen that the cave reflection characteristics are prominent on the seismic section, but there is also strong interference from the background formation reflections. Moreover, due to the imaging distance and seismic resolution, the reflection characteristics of faults and pores are not obvious, but these structures alter the amplitude values of the cave reservoir reflections, affecting the subsequent precise positioning of the reservoirs.

**Table 1:** Model parameters.

Number	Geology	Description	Velocity (m/s)
1	Stratum	Geological formation	4200
2	Stratum	Geological formation	4250
3	Pore	Diameter 8-15m	3700
4	Cave	Diameter 60-80m	3800
5	Fault	Separation 80-350m	3800
6	Cave	Diameter 82.5m	3700

**Fig 3:**
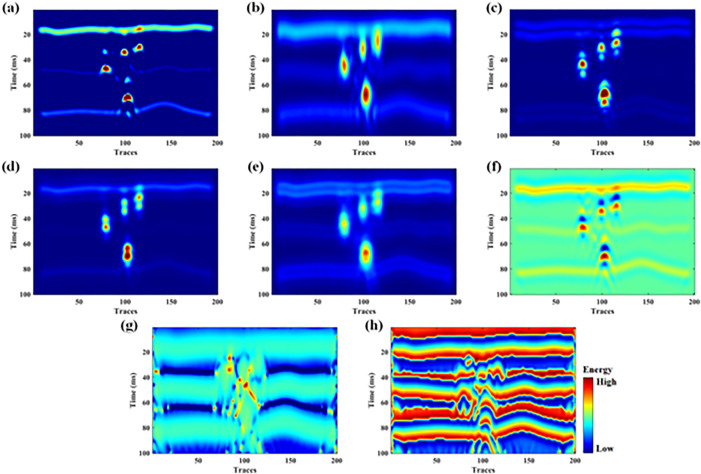
Seismic attributes of model data: (a) sweetness, (b) instantaneous amplitude, (c) structure tensor, (d) total energy, (e) gradient magnitude, (f) relative impedance, (g) instantaneous frequency, (h) instantaneous phase.

We conducted attribute extraction on the synthetic seismic data. The attributes involved in the calculation include sweetness, instantaneous amplitude, structure tensor, total energy, gradient magnitude, relative impedance, instantaneous frequency, instantaneous phase, as shown in Fig 3. Based on the consideration of bright spot characteristics on seismic profiles, data correlation, and processing efficiency, we have selected the following three most sensitive seismic attributes for cave reservoir identification: sweetness, instantaneous amplitude, and structure tensor. As shown in Figs 3a, 3b, and 3c respectively, these attributes are demonstrated in detail.

In light of the varying contributions of different attribute components to the final results, we conducted a comprehensive correlation analysis of each sensitive attribute prior to optimization and fusion. Since well data provides the only reliable means to verify subsurface structures, we established four pseudo-wells using randomly selected seismic traces (Traces 53, 99, 107, and 138). Given the seismic sampling interval of 0.5 ms, each trace contains 201 vertical sampling points. The cave reservoirs (labeled as 1) were identified through time-depth conversion of the velocity model, while other geological formations were classified as non-reservoirs (labeled as 2). This approach enabled us to systematically evaluate the correlation between seismic attributes and actual reservoir characteristics, ensuring the reliability of our subsequent attribute optimization and fusion process for cave reservoir identification.

**Fig 4:**
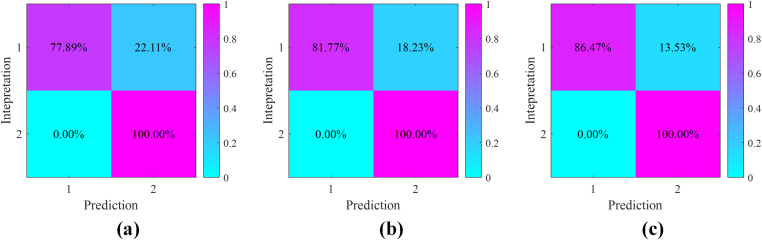
The confusion matrix of sensitive seismic attributes: (a) sweetness, (b) instantaneous amplitude and (c) structure tensor. Where tag 1 denotes cave reservoir, tag 2 denotes non-reservoir.

The prediction results of the pseudo-wells follow the following process: First, an optimal reservoir threshold is determined for each sensitive seismic attribute (sweetness, instantaneous amplitude, and structure tensor) through comprehensive analysis of their characteristic responses to cave reservoirs. This threshold determination takes into account both the statistical distribution of attribute values and their geological significance in reservoir identification. Second, precise reservoir separation is performed by applying these attribute-specific thresholds to delineate cave reservoirs from surrounding formations. It should be particularly noted that these thresholds are not fixed values but rather vary depending on: (1) the varying sensitivity and resolution of different attributes in characterizing cave reservoirs, and (2) the inherent differences in attribute dimensions and value ranges. This adaptive thresholding approach ensures more accurate and geologically meaningful reservoir prediction results while accounting for the unique characteristics of each seismic attribute in detecting and characterizing cave reservoirs. As shown in Fig 4, the confusion matrix of the pseudo-well interpretation results and the prediction results is presented. It can be seen that all three attributes effectively identified the non-reservoirs, but their performance in characterizing the cave reservoirs varies. The structure tensor attribute and the sweetness attribute have similar identification effects on the profile, but the structure tensor attribute achieves higher precision. After analysis, this is due to the phase difference in the “bright spots” highlighted by these two attributes, resulting in different precisions in cave location. Overall, all three attributes achieved effective identification of caves, but the precision needs to be further improved.

Based on the correlation of sensitive attributes indicated in Fig 4, we assigned initial fusion weights to each attribute. Following the quantitative Pearson correlation analysis between the pseudo-well reservoir indicators and the corresponding seismic attributes, the weights for sweetness, instantaneous amplitude, and structure tensor were set to 0.7789, 0.8177, and 0.8647, respectively. This process is guided by geological information, thus making our fusion knowledge-driven and statistically verifiable.

**Fig 5:**
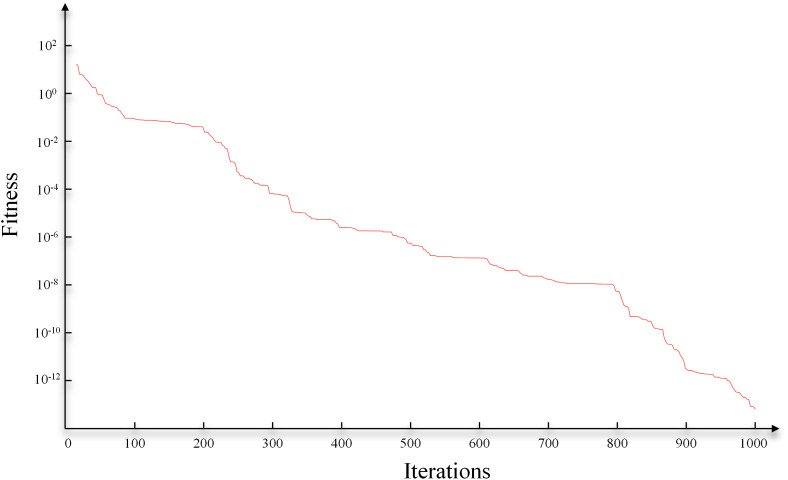
The iterations of GA.

Subsequently, to optimize the fusion parameters (optimal bandwidth), we employed the GA algorithm for this process. The search range for the optimization algorithm was set to [0.1,10], the initial solution was set to 5, and the number of iterations was set to 1000. Specifically, the population size was set to 200, with crossover and mutation probabilities of 0.8 and 0.05, respectively, ensuring a robust search for the optimal bandwidth within a reasonable computational timeframe. As shown in Fig 5, after 1000 iterations, the fitness function gradually stabilized and reached a lower level. Note that Fig 5 uses a logarithmic scale, making the later decline in fitness appear gradual. In reality, fitness is already low by 1000 iterations; further iterations yield negligible accuracy gains. This number balances precision and computational efficiency. The bandwidth parameter obtained at this point was 1.2, which we consider to be the optimal bandwidth parameter.

**Fig 6:**
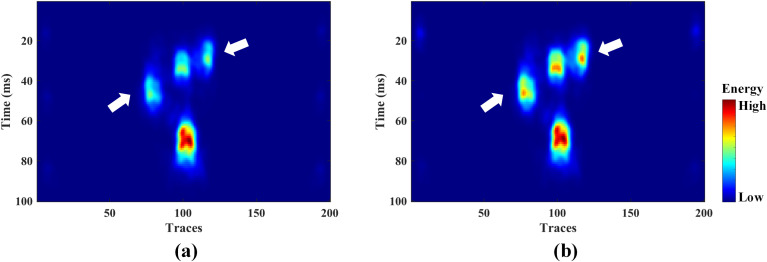
The fusion results: (a) PCA and (b) GA-KPCA.

Based on the optimal bandwidth parameters obtained, we further conducted multi-attribute fusion experiments using KPCA. For comparison, we also introduced the fusion results based on PCA. As shown in Fig 6, the attribute fusion results based on PCA and GA-KPCA are displayed respectively. The results demonstrate that after multi-attribute fusion, the non-reservoir information is effectively suppressed, and the cave reservoir characteristics are significantly highlighted. The fused profile exhibits superior performance compared to those obtained from single attributes. However, the fusion result based on GA-KPCA has a stronger ability to characterize caves with less obvious attribute differences. As indicated by the white arrows in the figure, the four sets of cave reservoirs are precisely identified.

**Fig 7:**
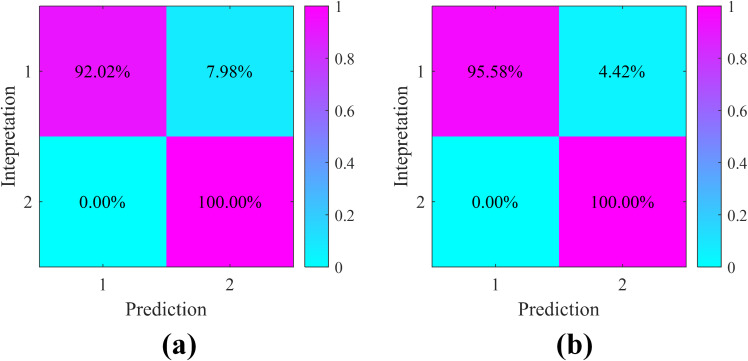
The confusion matrix: (a) PCA and (b) GA-KPCA.

To quantitatively assess the feasibility of the proposed method, we performed a correlation analysis of the results. As shown in Fig 7, we analyzed the confusion matrix of the pseudo-well data. The recognition accuracy of the cave reservoirs in the fusion results has been improved, but the improvement in accuracy based on GA-KPCA is more significant. The characterization accuracy of the cave reservoirs reaches 95.58%. The model data confirm the feasibility of the proposed GA-KPCA method in cave characterization.

To further investigate the sensitivity of the GA optimization to the reservoir threshold $T_{f}$, a comparative analysis was performed by varying $T_{f}$ from 0.2 to 0.8 with an interval of 0.1. As shown in the newly added sensitivity analysis in Table 2, the characterization accuracy remains relatively stable within the 0.4–0.6 range, confirming the robustness of the GA-KPCA method. However, when $T_{f}$ exceeds 0.7, the accuracy decreases by approximately 12% due to the loss of weak bead-like reflections from smaller caves. Conversely, a threshold below 0.3 increases the false positive rate by 10% as background geological noise is incorrectly identified as reservoirs. The convergence curves for different thresholds show that the GA consistently reaches a stable lower fitness level within 800 iterations, regardless of the initial threshold setting, although the absolute fitness value varies. This demonstrates that the optimal bandwidth obtained via GA is a reliable parameter that captures the intrinsic nonlinear features of the seismic data, provided the threshold is selected within a geologically reasonable window.

**Table 2:** Sensitivity analysis of characterization accuracy and convergence under different fusion thresholds.

Threshold	Characterization Accuracy (%)	Convergence Iterations
0.2	84.5	880
0.3	85.1	820
**0.4**	**95.6**	**770**
0.5	92.0	790
0.6	90.4	780
0.7	83.8	850
0.8	79.3	920

### 3.2. Field data

**Fig 8:**
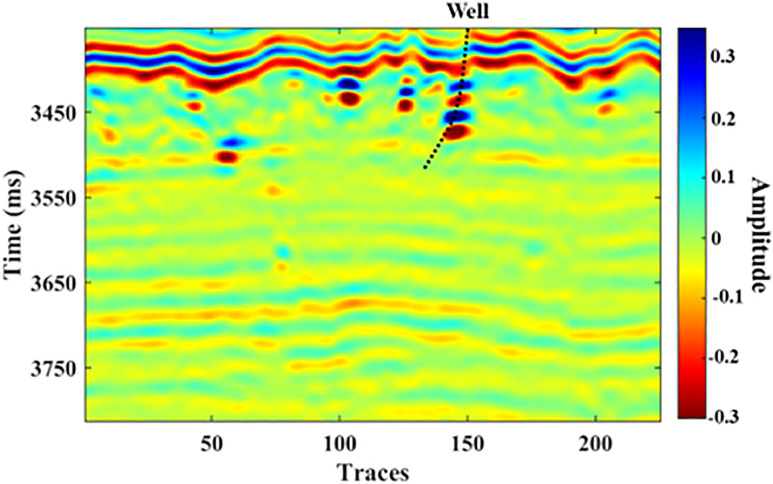
Field seismic data, the dashed black line denotes well trajectory.

We further utilized a field dataset to verify the effectiveness of the proposed GA-KPCA method. As shown in Fig 8, the data are from a certain area in the Tarim Basin, with the study formation being the Ordovician strata and the reservoir type being cave-type reservoirs, which aligns with the research objectives of this paper. The reservoir profile is characterized by bead-like reflections; however, due to the interference from strong reflective layers, the characterization of cave reservoirs based on seismic attributes is somewhat affected. Fig 8 also shows a well that encountered oil and gas in the target formation, which will be used as the validation well for subsequent effectiveness analysis.

**Fig 9:**
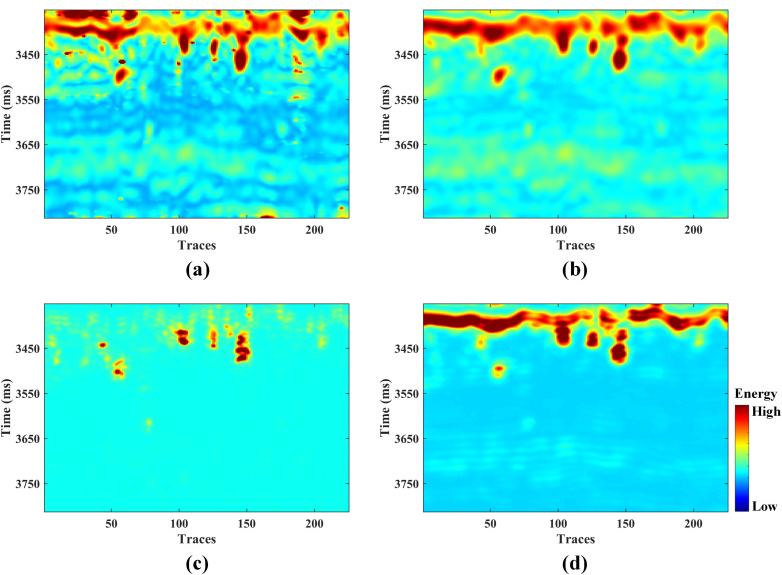
Sensitive seismic attributes of field data: (a) sweetness, (b) instantaneous amplitude, (c) structure tensor and (d) total energy.

Following the proposed algorithm’s workflow, we extracted the sensitive attributes from the actual seismic data. Based on the judgment of geological interpreters, we ultimately selected sweetness, instantaneous amplitude, structure tensor, and total energy as the sensitive seismic attributes. As shown in Fig 9, compared with the original seismic data in Fig 8, the seismic attributes have further enhanced the characterization of the bead-like cave reservoirs. However, the limitations of single attributes are also evident. Attributes such as sweetness and instantaneous amplitude effectively characterize the cave bodies, but the strong background reflection features have not been stripped away. The structure tensor attribute achieves separation of irrelevant background information, but there are deficiencies in the detailed characterization of the cave reservoirs. In summary, there is an urgent need to conduct fusion experiments. Based on the information from adjacent wells, we set the initial weights of the four fusion attributes mentioned above to 0.42, 0.51, 0.73, and 0.48, respectively.

**Fig 10:**
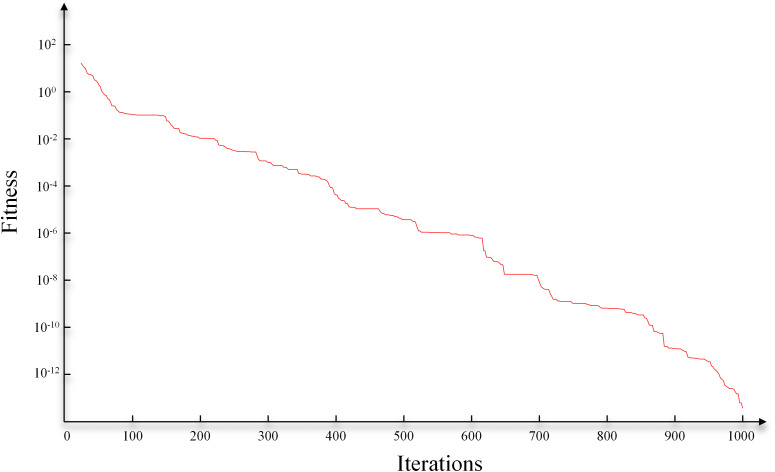
The iterations of GA for field data.

**Fig 11:**
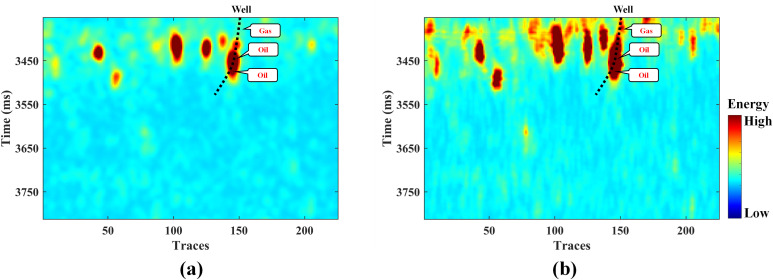
The fusion result of field data: (a) PCA and (b) GA-KPCA.

The hyperparameters of the GA algorithm were set consistent with those in the model test case. The optimization iteration process is shown in Fig 10. After 1000 iterations, the optimal bandwidth coefficient for GA-KPCA was determined to be 2.7. Subsequently, the KPCA fusion experiment was conducted based on this coefficient. For comparison, we also performed fusion using the PCA method. As shown in Fig 11, both methods effectively highlighted the cave bodies. However, the PCA method did not provide as rich structural detail as the GA-KPCA fusion result.

Moreover, according to the interpretation conclusions of the validation well, the well encountered one gas layer and two oil layers within the target formation’s cave reservoir. The GA-KPCA fusion method effectively characterized the hydrocarbon content of the cave reservoir, and achieved accurate reservoir identification. While the PCA fusion method failed to identify the gas layer, only two oil layers were identified, which could impact subsequent exploration and development processes. Consequently, the proposed method improves the identification success rate by 33% compared to the conventional PCA method. This metric is determined by the success in capturing the discrete hydrocarbon-bearing layers (one gas and two oil layers) encountered by the validation well.

While nonlinear supervised methods such as SVM or neural networks could theoretically serve as baselines, their practical application in this study area is limited by the sparsity of well-log data in the Ordovician strata. Supervised learners are prone to significant bias when the training samples are insufficient to represent the full heterogeneity of cave reservoirs. The GA-KPCA approach avoids this limitation by integrating geological knowledge with unsupervised kernel feature extraction, ensuring that the nonlinear characteristics are captured through an adaptive optimization of the bandwidth parameter rather than through complex training processes. This makes GA-KPCA more reliable for regional exploration where ground-truth labels are scarce. The comparative assessment using field data demonstrated the effectiveness and superiority of the proposed GA-KPCA method, indicating that this technique can be applied to characterize cave reservoirs.

While the proposed GA-KPCA method demonstrates superior accuracy in reservoir characterization, its computational efficiency requires careful consideration, particularly for large-scale 3D seismic applications. The current implementation involves an iterative optimization process guided by geological constraints, which, while ensuring high precision, increases computational demand compared to conventional PCA-based methods. In our case studies, the method was applied to 2D seismic profiles, achieving a practical balance between resolution and runtime. For field-scale 3D seismic interpretation, we recommend a hybrid workflow: (1) Use conventional attribute fusion or machine learning methods to rapidly identify potential reservoir zones across the 3D volume; (2) Apply the GA-KPCA optimization to key 2D sections or high-interest areas for detailed reservoir characterization, reducing uncertainty in critical zones.

This approach leverages the method’s strengths in precision while mitigating computational costs. Future improvements will focus on GPU-accelerated parallel computation and adaptive optimization strategies to enable efficient 3D implementation.

## 4. Conclusion

Aiming at the characterization of cave reservoirs, this paper proposes a cave reservoir characterization method based on GA-KPCA, which effectively addresses the inherent nonlinearity and subjectivity of conventional attribute fusion. By determining initial weights of each attribute according to geological interpretation conclusions, we provide a reliable geological basis that ensures the fusion results are grounded in reality rather than purely statistical correlations. Utilizing KPCA allows for the extraction of complex nonlinear features of cave data, while the GA algorithm adaptively optimizes key parameters to eliminate the uncertainty of manual selection.

In the tests using both model data and field data, the proposed GA-KPCA method demonstrated significant advantages, achieving a comprehensive capturing of the reservoir sequence and effectively overcoming the limitations of existing methods. This achievement not only provides a new and stable technical means for the exploration and development of cave reservoirs but also offers important theoretical support and practical guidance for subsequent related research.
